# Supplementary material for: Intestinal parasitic infections and associated factors among street dwellers and prison inmates: A systematic review and meta-analysis
Source: PLoS One. 2021 Aug 5;16(8):e0255641. doi: 10.1371/journal.pone.0255641 (PMC8341648; doi:10.1371/journal.pone.0255641)
Supplement: S1 File — (DOCX) [file pone.0255641.s001.docx]

-**Electronic search on PMC/Medline**

"Prevalence"[MeSH Terms] OR Burden[All Fields] AND "intestinal diseases, parasitic"[MeSH Terms] OR "opportunistic infections"[MeSH Terms] AND "risk factors"[MeSH Terms] OR associated factors[Title] AND (street[All Fields] AND dwellers[All Fields]) OR (Street[All Fields] AND Beggars[All Fields]) OR "prisoners"[MeSH Terms] OR "prison inmates"[All Fields] AND medline[sb]"

- **Explain why the search began in 2000**

The study groups in our study were not given much attention in previous years especially before 2000. So, we didn’t expect publications on these study groups before 2000. Let alone these groups, epidemiological information are scarce in study groups given much attention. Another reason is we wanted to analyze the published records in the past two decades on these study groups.

- **Date that the search was last conducted**

1^st^ December 2020.
