# Supplementary material for: Intestinal parasitic infections and associated factors among street dwellers and prison inmates: A systematic review and meta-analysis
Source: PLoS One. 2021 Aug 5;16(8):e0255641. doi: 10.1371/journal.pone.0255641 (PMC8341648; doi:10.1371/journal.pone.0255641)
Supplement: S2 File — (DOCX) [file pone.0255641.s002.docx]

| Source | Title | Selection | | | | Comparability | Outcome | | Quality |
| --- | --- | --- | --- | --- | --- | --- | --- | --- | --- |
|  |  | Representativeness  Of the sample | Sample size | Non-respondents | Ascertainment of exposure | The subject in different outcome groups are comparable | Assessment of the outcome | Statistical test |  |
| Ameya etal | Intestinal parasite infections and associated factors among inmates of Arba Minch prison, southern Ethiopia: cross sectional study | * | * | * | ** | * | ** | * | 9 |
| Angal etal | Determining intestinal parasitic infections (IPIs) in inmates from Kajang Prison, Selangor, Malaysia for improved prison management | * | * | * | ** | * | ** | * | 9 |
| Mardu etal | Prevalence of intestinal parasites and associated risk factors among inmates of Mekelle prison, Tigrai Region, Northern Ethiopia, 2017 | * | * | * | ** | * | ** | * | 9 |
| Terefe etal | Intestinal helminth infections among inmates in Bedele prison with emphasis on soil-transmitted helminths | * | * | * | * | * | ** | * | 8 |
| Shrestha etal | Intestinal Parasitic Infections among Prison Inmates in Kathmandu Nepal | * | * | * | * | * | ** | * | 8 |
| Amit etal | Prevalence of intestinal parasites and urinary pathogens among prison inmates in central jail of Bhopal (MP) | * | - | * | * | * | ** | * | 7 |
| Ahmed et al | Passive surveillance of communicable diseases among inmates of Jos central prison, Nigeria | - | * | * | * | * | ** | * | 7 |
| Nadabo etal | Status of intestinal parasites in inmates of a correctional facility, Jos, Nigeria | * | * | - | ** | * | ** | - | 7 |
| Mamo | Intestinal Parasitic Infections among Prison Inmates and Tobacco Farm Workers in Shewa Robit, North-Central Ethiopia | * | * | * | ** | * | ** | * | 9 |
| Curval etal | Prevalence of intestinal parasites among inmates in Midwest Brazil | * | * | * | ** | * | ** | * | 9 |
| Rob etal | Risk factors associated with intestinal parasitic infections among inmates of Kisii prison, Kisii county, Kenya | * | * | * | ** | * | ** | * | 9 |
| Feleke etal | Intestinal parasitic infections and associated factors among street dwellers’ in Dessie town, North-East Ethiopia: a cross sectional study | * | * | * | ** | * | ** | * | 9 |
| Bailey etal | Factors associated with parasitic infection  amongst street children in orphanages across  Lima, Peru | * | * | - | ** | * | ** | * | **7** |
| Mekonnen etal | Prevalence of Intestinal Parasitic Infections and Related Risk Factors among Street Dwellers in Addis Ababa, Ethiopia | * | * | * | * | * | ** | * | 8 |
| Kheir etal | Prevalence of intestinal parasites, associated risk factors and social background of street children in Khartoum State, Sudan | * | * |  | * | * | ** | * | **7** |
| Zenu etal | Prevalence of intestinal parasitic infections and associated factors among street children in Jimma town; south West Ethiopia in 2019: a cross sectional study | * | * | * | ** | * | ** | * | 9 |
| Lakew etal | Prevalence of intestinal parasites among street beggars in Jimma town, Southwest Ethiopia | * | * |  | * | * | ** | * | **7** |
